# AI-Powered Clinical Documentation and Clinicians’ Electronic Health Record Experience: A Nonrandomized Clinical Trial

**DOI:** 10.1001/jamanetworkopen.2024.32460

**Published:** 2024-09-06

**Authors:** Tsai-Ling Liu, Timothy C. Hetherington, Casey Stephens, Andrew McWilliams, Ajay Dharod, Tracey Carroll, Jeffrey A. Cleveland

**Affiliations:** 1 Center for Health System Sciences, Atrium Health, Charlotte, North Carolina; 2 Advanced Analytics, Atrium Health, Charlotte, North Carolina; 3 Department of Internal Medicine, Atrium Health, Charlotte, North Carolina; 4 Medical Informatics, Atrium Health, Charlotte, North Carolina; 5 Informatics and Analytics, Department of Internal Medicine, Wake Forest University School of Medicine, Winston-Salem, North Carolina; 6 General Internal Medicine, Department of Internal Medicine, Wake Forest University School of Medicine, Winston-Salem, North Carolina; 7 Department of Implementation Science, Division of Public Health Sciences, Wake Forest University School of Medicine, Winston-Salem, North Carolina; 8 Wake Forest Center for Healthcare Innovation, Wake Forest University School of Medicine, Winston-Salem, North Carolina; 9 Wake Forest Center for Artificial Intelligence, Wake Forest University School of Medicine, Winston-Salem, North Carolina; 10 Information Technology, Atrium Health Wake Forest Baptist Health, Winston-Salem, North Carolina; 11 Department of Pediatrics, Atrium Health, Charlotte, North Carolina

## Abstract

This nonrandomized clinical trial investigated the electronic health record (EHR) experiences of clinicians before and after implementation of an artificial intelligence (AI)–powered clinical documentation tool.

## Introduction

In health care, artificial intelligence (AI)–powered clinical documentation tools aim to reduce physician burnout, optimize workflows, and refine the accuracy of clinician documentation.^1,2,3,4^ Some of these AI tools can generate a preliminary clinical note by listening to the interaction between a clinician and a patient, then synthesizing the conversation into a draft clinical note. We evaluated clinicians’ experiences with clinical documentation before and after implementing an AI-powered clinical documentation tool.

## Methods

This nonrandomized clinical trial recruited family medicine, internal medicine, and general pediatrics clinicians (physicians and advanced practice practitioners) from all outpatient clinics in North Carolina and Georgia within Atrium Health. Clinicians who accepted the invitation were the intervention group and were divided into 4 waves between June and August 2023 based on clinic locations (n = 112). Clinicians received a 1-hour in-person training on the AI-powered clinical documentation tool used in this study, Dragon Ambient eXperience (DAX) Copilot (Nuance), before activation of their accounts. A comparison group of clinicians not using this study’s AI clinical documentation tool (n = 117) was assembled through 2 approaches with the goal of identifying similar practice locations and specialties to the intervention group: (1) service line leaders identified clinicians and encouraged them to participate as controls; and (2) clinicians who expressed initial interest in the AI clinical documentation tool, but later opted out after the informational meetings, were invited to participate as controls. The power calculation for 2 proportions in a repeated measures design indicates that 83 participants in the intervention group and 55 participants in the control group would provide 80% power to detect differences between the 2 groups. The trial protocol (Supplement 1) was exempted by the Wake Forest University School of Medicine institutional review board and granted waiver of informed consent because no protected health information would be used or disclosed. The study followed the TREND reporting guideline (eFigure in Supplement 2).

A 7-question survey was created in REDCap using a subset of questions from the American Medical Association Organizational Biopsy electronic health record (EHR)–specific questions (eTable in Supplement 2)^5^; the survey was anonymously emailed to 230 participants before and 5 weeks after the intervention implementation. Each question was assigned a code ranging from 1 to 5, with 1 representing the worst response and 5 representing the best response. If a respondent’s score increased from the presurvey to the postsurvey, we categorized the question as decreased time and/or improved experience. Conversely, if the score decreased, we labeled it as increased time and/or worse experience. When the score remained the same in both pre- and postsurveys, we categorized it as unchanged. χ^2^ test was used to compare responses between the 2 groups using SAS version 9.4 (SAS Institute). Two-sided *P* < .05 was considered statistically significant.

## Results

Among 140 respondents, 80 (57.1%) were female, 55 (39.3%) had 5 to 15 years of experience, and 52 (37.1%) had 15 to 25 years of experience (Table). In the intervention group, 40 of 85 (47.1%) reported decreased time on the EHR at home (vs 8 of 55 [14.5%] in the control group; *P* < .001) and 38 of 85 (44.7%) reported decreased weekly time on the EHR outside normal work hours (vs 11 of 55 [20.0%] in the control group; *P* = .003). Moreover, 37 of 85 intervention respondents (43.5%) reported decreased time on documentation after the visits (vs 10 of 55 [18.2%] in the control group; *P* = .002) and 38 of 85 (44.7%) reported less frustration using the EHR (vs 8 of 55 [14.5%] in the control group; *P* < .001). Conversely, a mean (SD) of 44.7% (1.7) of participants in the intervention group and 68.7% (2.3) in the control group reported that their EHR experiences were comparable before and after the intervention (Figure).

**Table.** Demographics Among Survey Respondents

Characteristics	Respondents, No. (%)		
	Intervention (n = 85)	Control (n = 55)	*P* value^a^
Gender^b^			
Male	43 (50.6)	17 (30.9)	.02
Female	42 (49.4)	38 (69.1)	
Experience, y			
<5	10 (11.8)	5 (9.1)	.87
5-15	32 (37.6)	23 (41.8)	
15-25	33 (38.8)	19 (34.5)	
>25	10 (11.8)	8 (14.5)	

^a^ χ^2^ test was used to compare responses between the 2 groups.

^b^ Self-reported gender.

**Figure.**
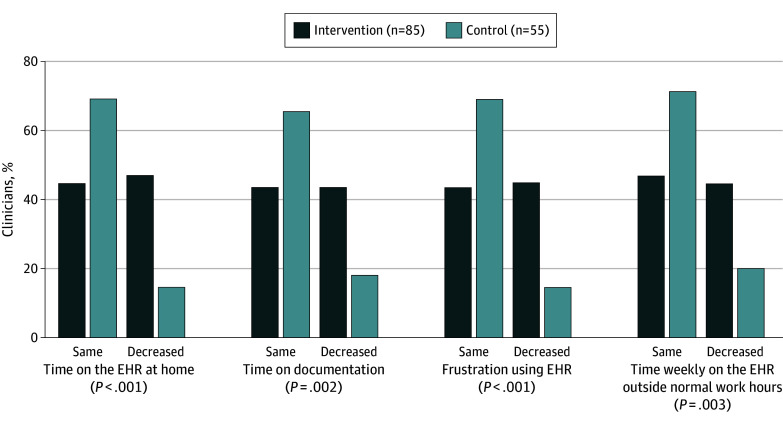
Survey Responses About Electronic Health Record (EHR) Experience Before and After Intervention

## Discussion

Approximately half of clinicians using the AI-powered clinical documentation tool based on interest reported a positive outcome, potentially reducing burnout. However, a significant subset did not find time-saving benefits or improved EHR experience. Study limitations include potential selection bias and recall bias in both groups. Further research is needed to identify opportunities for improvement and understand the impact on different clinician subsets and health systems.

## Notes

Supplement 1.
Trial Protocol

Supplement 2. **eTable.** DAX Copilot Provider Feedback Survey **eFigure.** Participant Selection Flowchart

Supplement 3.
Data Sharing Statement
