# Supplementary material for: AI-Powered Clinical Documentation and Clinicians’ Electronic Health Record Experience: A Nonrandomized Clinical Trial
Source: JAMA Netw Open. 2024 Sep 6;7(9):e2432460. doi: 10.1001/jamanetworkopen.2024.32460 (PMC11380097; doi:10.1001/jamanetworkopen.2024.32460)
Supplement: Supplement 1. — Trial Protocol [file jamanetwopen-e2432460-s001.pdf]

Study Title: Dax Express Pilot Evaluation

Principal Investigator, Co-investigator(s): Tsai-Ling Liu (co-PI), Timothy C Hetherington (co-PI), Andrew McWilliams, Marc Kowalkowski, Yhenneko J Taylor, Nicholas M. Pajewski, Ajay Dharod, Richa Bundy

Sponsor or funding source: Atrium Health/Wake Forest internal funding

Background: Nuance is a company owned by Microsoft that has provided our dictation software and hardware for many years (with Cerner and now Epic). Their tried-and-true platform is a microphone (called the 'dragon') connected via a cord to nearly every desktop computer. Providers sit at the computer and dictate notes into the 'dragon'. The speech to text software then puts the note into Epic. Ability to do this in whatever area of Epic that will accept text.

This technology has advanced to allow for ambient listening to conversations between patient and provider in the actual exam room. The speech-to-text then parses the conversation and generates a note. Currently, that automated note is reviewed by a nuance employee and then sent to the provider. This product is called 'Dax' and is being piloted with 10 orthopedic providers at Wake Forest.

More recently Nuance developed a fully automated version of Dax called 'Dax Express'. Dax Express uses AI to generate the provider note in real-time (without human review/editing). It also elevates accuracy and structure with an integrated GPT model.

Atrium Health will be the first test site in the world for Dax Express. Atrium health is being given licenses for the pilot until the end of the year; thus leaders hope to start the pilot in June.

Rationale: The rationale for a rigorous evaluation in collaboration with academics include:

1. This is new/innovative largely untested and unpublished.
2. Likely potential for further industry or federal (AHRQ) funding as there are potential huge efficiency gains.
3. Huge expense and thus a weighty decision for clinical operations, if they choose to proceed for all providers.

Study Design: A non-randomized stepped-wedge trial assessing provider satisfaction, patient experience, and provider efficiency.

Study Population: Providers who have expressed interest in participating in the Dax Express Pilot Evaluation. Not all providers will be provisioned access to Dax Express. The operational team has pre-determined the list of providers who will receive access to Dax Express. Geographic location was considered to ease the burden of training providers. The remaining providers will participate in the control group.

A over 200 providers have volunteered to participate. Only 100-150 will be provisioned access to Dax Express as part of the pilot in 2023. The remaining providers can serve as a control across the whole period. The providers will be onboarded in waves (based on geographic proximity) every few weeks to a month. Those awaiting roll out can contribute control time before flipping to the intervention group.

Outcome Measurements: The primary outcomes including providers interacted with Dax Express as

intervention group and providers with standard care as comparison group. Provider characteristics, including providers' age, gender, race/ethnicity, years of experience, provider specialty, and monthly encounter/patient volume, will be included as covariates in the final analytic models.

1. Provider metrics: Epic Clarity data and Epic provider data (e.g., user action logs) will generate reports containing days with appointments, visit volumes, progress note length, time in notes per 8 hour scheduled day, time in system per 8 hour scheduled day, work outside of work, percentages of visits closed in same day. Length of provider time will be extracted and calculated based on these reports. Additional provider metrics may be included if recommended by subject matter experts.
2. Operational metrics: Finance and performance improvement teams will generate reports on weekly visit volumes, revenues, work revenue per FTE, gross revenue per visit, level codes, accounting for time in office. Additional operational metrics may be included if recommended by the finance and performance improvement teams.
3. Pre- and post- provider satisfaction: a baseline survey will be sent to participating providers before the launch of DAX Express. A follow-up provider satisfaction survey will be sent after 5 weeks of the launch. There will be a link on the surveys directing providers to the vendor survey to complete if they choose to do so.
4. Patient satisfaction with provider interactions: patient satisfaction surveys are collected as part of routine care, which will be accessed through Medallia.
5. DAX utilization: we will get number of visits used DAX Express and number of notes copied into Epic from the vendor (Nuance).

<u>**Statistical Analysis**</u>**:** Sample characteristics will be reported as medians or percentages as appropriate. Descriptive statistics, two-sample t-test, interrupted time series (ITS), and generalized linear model will be used to examine the distributions of outcomes (i.e., provider metrics, operational metrics, DAX utilization, patient satisfaction, and provider satisfaction) at pre- and post- intervention (i.e., launch of DAX Express). Sub-analysis on stratifying DAX users into active, lapsed, and dropped users will also be analyzed using the same models. Details listed in Table 1.

Table 1. Outcomes measures and analysis summary

Outcome	Measure	Granularity	Analysis
**Provider metrics**	days with appointments, weekly visit volumes, progress note length, time in notes per 8 hour scheduled day, time in system per 8 hour scheduled day, work outside of work, percentages of visits closed in same day, etc.	Weekly<br><br>Daily	Interrupted time series<br><br>Generalized linear model
**Operational metrics**	weekly visit volumes, revenues, accounting for time in office<br><br>work revenue per FTE, gross revenue per visit, level codes, accounting for time in office, etc.	Weekly<br>Daily<br><br>Monthly<br>Daily	Interrupted time series<br>Generalized linear model<br>Two-sample t-test<br>Generalized linear model
**Provider satisfaction**	Pre- and post-surveys	N/A	Two-sample t-test,

			generalized linear model
**Patient satisfaction**	Pre- and post-surveys	N/A	Two-sample t-test
**DAX utilization**	number of visits used DAX Express and number of notes copied into Epic	Weekly<br>Daily	Interrupted time series<br>Generalized linear model

66

Human Subjects Protection

Subject Recruitment Methods

A over 200 providers have volunteered to participate. Only 100-150 will be a part of the pilot in 2023, with the remainder being the first to go in 2024. This latter group can serve as a control across the whole period. The remaining providers will be onboarded in waves (based on geographic proximity) every few weeks to a month. Those awaiting roll out can contribute control time before flipping to the intervention group.

Informed Consent

Written informed consent will not be obtained. The risk of harm or discomfort that may occur as a result of taking part in this research study is not expected to be more than in daily life or from routine physical or psychological examinations or tests. The rights and welfare of study will be protected through the use of measures to maintain the confidentiality of study information. Study results will be presented or published in lieu of providing individual subjects additional information regarding the study.

Confidentiality and Privacy

Confidentiality will be protected by collecting only information needed to assess study outcomes, minimizing to the fullest extent possible the collection of any information that could directly identify subjects, and maintaining all study information in a secure manner. To help ensure subject privacy and confidentiality, only a unique study identifier will appear on the data collection form. Any collected patient identifying information corresponding to the unique study identifier will be maintained on a linkage file, store separately from the data. The linkage file will be kept secure, with access limited to designated study personnel. Following data collection subject identifying information will be destroyed *three years after closure of the study,* consistent with data validation and study design, producing an anonymous analytical data set. Data access will be limited to study staff. Data and records will be kept locked and secured, with any computer data password protected. No reference to any individual participant will appear in reports, presentations, or publications that may arise from the study.

Data and Safety Monitoring

The principal investigator will be responsible for the overall monitoring of the data and safety of study participants. The principal investigator will be assisted by other members of the study staff.

Reporting of Unanticipated Problems, Adverse Events or Deviations

Any unanticipated problems, serious and unexpected adverse events, deviations or protocol changes will be promptly reported by the principal investigator or designated member of the research team to the IRB and sponsor or appropriate government agency if appropriate.

Appendix

1. Pre- and post- provider satisfaction survey (for both intervention and control group)
2. Survey on provider’s satisfaction with the software provided by the vendor

Appendix 1. Pre- and post- provider satisfaction survey (for both intervention and control group)

DAX Express Provider Feedback Survey

A A A

Dear Colleagues,

Thank you for participating in the Dragon Ambient eXperience (DAX) Express pilot. As we have discussed, we are piloting DAX Express to see how well it improves the time physician and APPs spend on documentation in the Electronic Health Record.

To help understand the usefulness of DAX Express, we are conducting a brief survey before and after the pilot. The attached 7 question survey is an extract from the AMA Organizational Biopsy focused on use of technology.

Prior to starting the pilot, please respond to these questions with your current/historical experience to establish a baseline.

Thank you!

Jeff Cleveland

1. The amount of time I spend on the Electronic Medical Record (EMR) at home is:

- ○ Minimal/None
- ○ Modest
- ○ Satisfactory
- ○ Moderately High
- ○ Excessive

reset

2. The amount of time I spend on documentation is:

- ○ Optimal
- ○ Good
- ○ Satisfactory
- ○ Marginal
- ○ Poor

reset

3. The EMR adds to the frustration of my day.

- ○ Strongly Disagree
- ○ Disagree
- ○ Neither Agree or Disagree
- ○ Agree
- ○ Strongly Agree

reset

4. How much time WEEKLY do you spend on the EMR outside your normal work hours?

- ◯ 0-2 hours
- ◯ 2-4 hours
- ◯ 4-6 hours
- ◯ 6-8 hours
- ◯ More than 8 hours

reset

5. What is the primary way you document clinical information?

- ◯ I type my own notes and/or use templates.
- ◯ Team documentation
- ◯ Dictation or transcriptionist
- ◯ Dictation to voice recognition
- ◯ A hybrid of templates, typing and voice recognition
- ◯ Other

reset

6. What is your sex?

- ◯ Female
- ◯ Male
- ◯ Non-binary
- ◯ Prefer not to answer

reset

7. What are your years of experience (post-training)?

- ◯ < 5 years
- ◯ 5-15 years
- ◯ 15-25 years
- ◯ 25+ years

reset

Submit

Appendix 2. Survey on provider's satisfaction with the software provided by the vendor

NUANCE

Your experience with DAX Express

We would like your feedback about DAX Express. Your input helps us improve the product experience. The following survey typically takes less than 5 minutes. We will use the below information only to contact you for research and product support purposes.

First Name

Last name

Work email address

How satisfied or dissatisfied are you with your current work-life balance? (select one)

- ○ Very satisfied
- ○ Somewhat satisfied
- ○ Neither satisfied nor dissatisfied
- ○ Somewhat dissatisfied
- ○ Very dissatisfied

Rate your overall experience being trained and supported using DAX Express (select one)

- ◯ Very positive
- ◯ Somewhat positive
- ◯ Neither positive nor negative
- ◯ Somewhat negative
- ◯ Very negative

Please rate your overall experience with the in-app training that appeared when you first logged into DAX Express. (select one)

- ◯ Very positive
- ◯ Somewhat positive
- ◯ Neither positive nor negative
- ◯ Somewhat negative
- ◯ Very negative
- ◯ Not applicable / not sure

What could we do to better train or support you to use DAX Express effectively? (enter text, optional)

Briefly describe how DAX Express has impacted your practice. (enter text)

How disappointed would you be if you didn't have access to DAX Express? (select one)

- ◯ Not disappointed
- ◯ Somewhat disappointed
- ◯ Very disappointed
- ◯ Not sure

Have you experienced improvements in any of the following with DAX Express? (select one per statement)

	Yes	No
Burnout or fatigue	◯	◯
Experience documenting	◯	◯
Experience providing care	◯	◯
Documentation quality (e.g., accuracy, detail, consistency)	◯	◯
Other (please specify) ____	◯	◯

To what extent did DAX Express impact the time you spend on clinical documentation? (select one)

- ◯ I spend more time
- ◯ I spend the same amount of time
- ◯ I spend less time
- ◯ I'm not sure
