# Supplementary material for: AI-Powered Clinical Documentation and Clinicians’ Electronic Health Record Experience: A Nonrandomized Clinical Trial
Source: JAMA Netw Open. 2024 Sep 6;7(9):e2432460. doi: 10.1001/jamanetworkopen.2024.32460 (PMC11380097; doi:10.1001/jamanetworkopen.2024.32460)
Supplement: Supplement 2. — eTable. DAX Copilot Provider Feedback Survey eFigure. Participant Selection Flowchart [file jamanetwopen-e2432460-s002.pdf]

Supplemental Online Content

Liu TL, Hetherington TC, Stephens C, et al. AI-powered clinical documentation and clinicians' electronic health records experience: a nonrandomized clinical trial. *JAMA Netw Open*. 2024;7(9):e2432460. doi:10.1001/jamanetworkopen.2024.32460

eTable. DAX Copilot Provider Feedback Survey
eFigure. Participant Selection Flowchart

This supplemental material has been provided by the authors to give readers additional information about their work.

© 2024 Liu TL et al. *JAMA Network Open*.

DAX Copilot Provider Feedback Survey

A A A

Dear Colleagues,

Thank you for participating in the Dragon Ambient eXperience (DAX) Copilot. As we have discussed, we are piloting DAX Copilot to see how well it improves the time physicians and APPs spend on documentation in the Electronic Health Record.

To help understand the usefullness of DAX Copilot, we are conducting a brief survey before and after the pilot. The attached 7 question survey is an extract from the AMA Organizational Biopsy focused on the use of technology.

Priot to starting the pilot, please respond to these questions with your current/historical experience to establish a baseline.

Thank you!

Jeff Cleveland

1. The amount of time I spend on the Electronic Medical Record (EMR) at home is:

- ○ Minimal/None
- ○ Modest
- ○ Satisfactory
- ○ Moderately High
- ○ Excessive

2. The amount of time I spend on documentation is:

- ○ Optimal
- ○ Good
- ○ Satisfactory
- ○ Marginal
- ○ Poor

© 2024 Liu TL et al. *JAMA Network Open*.

3. The EMR adds to the frustration of my day.

- ◯ Strongly Disagree
- ◯ Disagree
- ◯ Neither Agree or Disagree
- ◯ Agree
- ◯ Strongly Agree

4. How much time WEEKLY do you spend on the EMR outside your normal work hours?

- ◯ 0-2 hours
- ◯ 2-4 hours
- ◯ 4-6 hours
- ◯ 6-8 hours
- ◯ More than 8 hours

5. What is the primary way you document clinical information?

- ◯ I type my own notes and/or use templates.
- ◯ Team documentation
- ◯ Dictation or transcriptionist
- ◯ Dictation to voice recognition
- ◯ A hybrid of templates, typing and voice recognition
- ◯ Other

6. What is your sex?

- ◯ Female
- ◯ Male
- ◯ Non-binary
- ◯ Prefer not to answer

7. What are your years of experience (post-training)?

- ◯ < 5 years
- ◯ 5-15 years
- ◯ 15-25 years
- ◯ 25+ years

© 2024 Liu TL et al. *JAMA Network Open*.

Submit

Powered by REDCap

eFigure. Participant Selection Flowchart

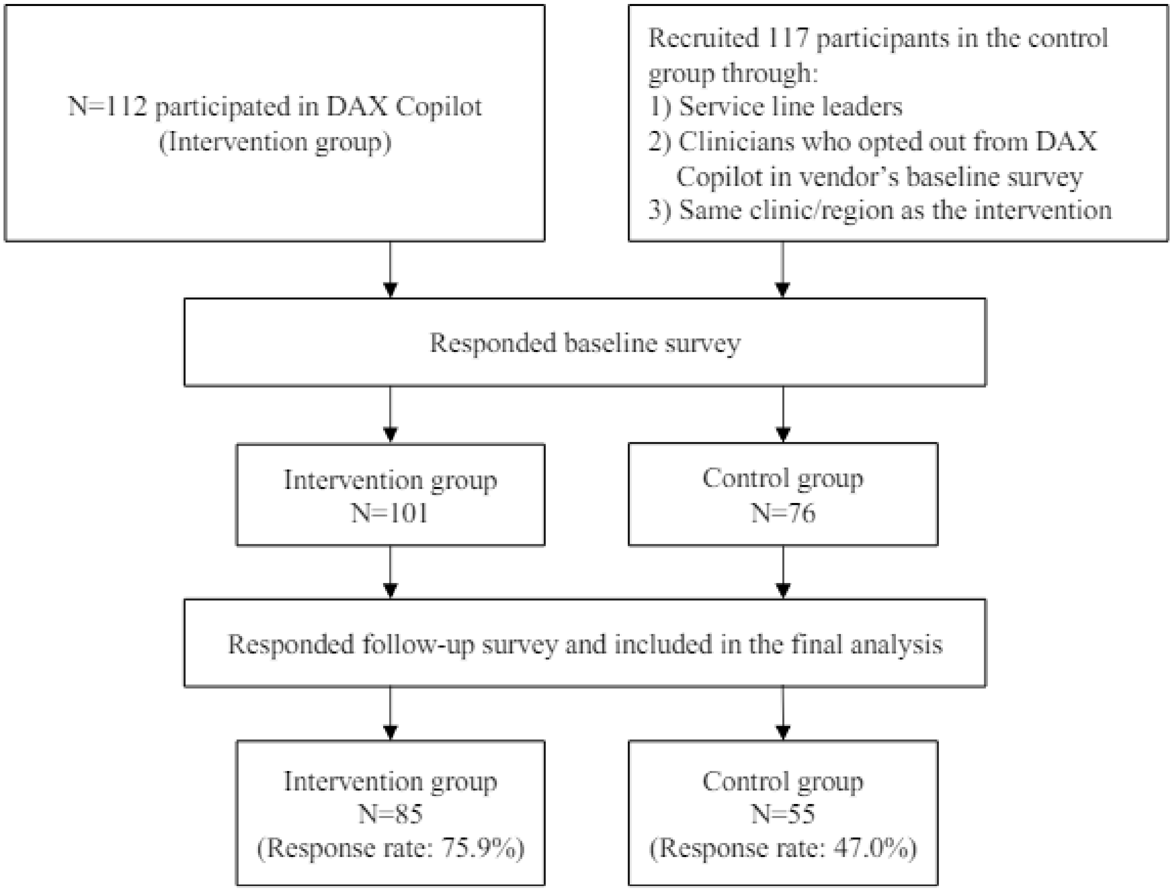

© 2024 Liu TL et al. *JAMA Network Open*.
