# Supplementary material for: AI-Powered Clinical Documentation and Clinicians’ Electronic Health Record Experience: A Nonrandomized Clinical Trial
Source: JAMA Netw Open. 2024 Sep 6;7(9):e2432460. doi: 10.1001/jamanetworkopen.2024.32460 (PMC11380097; doi:10.1001/jamanetworkopen.2024.32460)
Supplement: Supplement 3. — Data Sharing Statement [file jamanetwopen-e2432460-s003.pdf]

Data Sharing Statement

Liu. AI-Powered Clinical Documentation and Clinicians' Electronic Health Records Experience. *JAMA Netw Open*. Published September 06, 2024. doi:10.1001/jamanetworkopen.2024.32460

Data
Data available: No
